# A large-scale forward genetic screen for maize mutants with altered lignocellulosic properties

**DOI:** 10.3389/fpls.2023.1099009

**Published:** 2023-03-07

**Authors:** Shaogan Wang, Stefan Robertz, Merve Seven, Florian Kraemer, Benjamin M. Kuhn, Lifeng Liu, China Lunde, Markus Pauly, Vicente Ramírez

**Affiliations:** ^1^ Institute for Plant Cell Biology and Biotechnology-Cluster of Excellence on Plant Sciences, Heinrich Heine University Düsseldorf, Düsseldorf, Germany; ^2^ Department of Plant and Microbial Biology, Energy Biosciences Institute, University of California, Berkeley, Berkeley, CA, United States; ^3^ Plant Gene Expression Center, Agricultural Research Service, U.S. Department of Agriculture, Albany, CA, United States

**Keywords:** maize, cell wall, mutant, lignocellolusic biomass, saccharification

## Abstract

The development of efficient pipelines for the bioconversion of grass lignocellulosic feedstocks is challenging due to the limited understanding of the molecular mechanisms controlling the synthesis, deposition, and degradation of the varying polymers unique to grass cell walls. Here, we describe a large-scale forward genetic approach resulting in the identification of a collection of chemically mutagenized maize mutants with diverse alterations in their cell wall attributes such as crystalline cellulose content or hemicellulose composition. Saccharification yield, i.e. the amount of lignocellulosic glucose (Glc) released by means of enzymatic hydrolysis, is increased in two of the mutants and decreased in the remaining six. These mutants, termed *candy-leaf* (*cal)*, show no obvious plant growth or developmental defects despite associated differences in their lignocellulosic composition. The identified *cal* mutants are a valuable tool not only to understand recalcitrance of grass lignocellulosics to enzymatic deconstruction but also to decipher grass-specific aspects of cell wall biology once the genetic basis, i.e. the location of the mutation, has been identified.

## Introduction

Lignocellulosic biomass represents most of the carbon-based photosynthetic products fixed by land plants. As an abundant and sustainable feedstock, plant lignocellulosic biomass can be used to produce environment-friendly energy and other high-added value chemicals (Himmel et al., 2007; Pauly and Keegstra, 2010; Somerville et al., 2010). Grass species from the Poaceae family such as maize, sorghum, miscanthus, or switchgrass have been identified as promising feedstocks due to their fast growth and the large amounts of fermentable sugars locked in their cell walls in the form of a complex network of carbohydrate and other polymers (Lewandowski et al., 2000; Pauly and Keegstra, 2008; Schmer et al., 2008).

Grass cell walls are mainly composed of cellulose, glucuronoarabinoxylan, β-(1,3;1,4)-glucans, and lignin (reviewed in Vogel, 2008). Cellulose is the most common and abundant polysaccharide in both primary and secondary grass cell walls. Cellulose consists of linear β-(1,4)-linked glucan chains aggregated into microfibrils with crystalline and partially amorphous structural features (Harris et al., 2012; Marriott et al., 2016; Rongpipi et al., 2018).

Glucuronoarabinoxylan (GAX) is the predominant non-cellulosic polysaccharide in both primary and secondary cell walls of grasses (Scheller and Ulvskov, 2010; Pauly et al., 2013). The typical structure of grass GAX consists of a linear xylan backbone formed by β-1,4-linked D-xylopyranose residues, which can be methylated, acetylated or further substituted with mono- and/or oligosaccharides composed of xylose, arabinose (Ara), galactose (Gal) and uronic acid residues (Teleman et al., 2000; Kabel et al., 2003; Pauly et al., 2013; Peña et al., 2016; Tryfona et al., 2019). The majority of the GAX molecules display a three-fold screw conformation due to the arabinosyl substitutions and interact with amorphous cellulose. In contrast, in softwoods and dicot plants xylan acquires a two-fold screw conformation and interacts mainly with crystalline cellulose (Simmons et al., 2016; Terrett et al., 2019b; Gao et al., 2020).

β-(1,3;1,4)-glucans, also termed mixed-linked glucans (MLG), are unbranched glucans composed of β-(1,4)-linked cellotriosyl and cellotetrasyl units interspersed with β-(1,3)-glucosyl linkages. The presence of MLG is a hallmark of grass cell walls and is usually not present in dicot species or non-commelinid monocots (reviewed by Burton and Fincher, 2009). MLG is highly accumulated in primary walls of rapidly growing tissues, but it can also be found in secondary walls in mature organs (Fincher and Stone, 2004; Vega-Sanchez et al., 2012; Vega-Sanchez et al., 2013). Although the functional role of MLG in grasses is under debate, it has been hypothesized that it might be involved in energy-storage and cell wall strengthening or reinforcement (Carpita et al., 2001; Burton and Fincher, 2009; Vega-Sanchez et al., 2012; Smith-Moritz et al., 2015).

Lignin is a large polyphenolic compound built *via* polymerization of various monolignols which makes up 20% of the dry weight of the grass secondary cell wall (Marriott et al., 2016; Ralph et al., 2019). Ferulic acid (FA) residues can be attached *via* ester linkages to the α-1,3-arabinosyl residues of grass GAX, and ferulated xylan further connects with monolignols thus growing lignin polymers by enzymatic radical coupling reactions (de Oliveira et al., 2015; Terrett and Dupree, 2019; Eugene et al., 2020). The covalent cross-link of lignin to GAX forms a matrix network in which cellulose microfibrils are embedded, enhancing the hydrophobicity and rigidity of the cell wall.

The most important aspect of an economical competitive feedstock is a high production of plant biomass per field area (Tilman et al., 2006; Pauly and Keegstra, 2008). However, also the bioconversion of the feedstock should be accomplished with as little energy input as possible. Such a bioconversion potential of a given lignocellulosic biomass feedstock is often determined by its saccharification yield, i.e., how efficient is the release of sugars by means of enzymatic hydrolysis (McCann and Carpita, 2015; Zoghlami and Paes, 2019). During saccharification, enzymatic cocktails harboring diverse hydrolase activities break down the wall carbohydrates releasing monosaccharides - mainly glucose (Glc) and xylose (Xyl) - and small amounts of other soluble di- and oligo-saccharides. Unfortunately, the plant wall is naturally recalcitrant to enzymatic hydrolysis not only due to a heterogeneous carbohydrate composition, but also due to the complex interactions among the diverse polymeric components. One challenge to efficiently utilize lignocellulosics from grass feedstocks is the limited understanding of its synthesis, *in muro* architecture, and degradation of the wall components and how the diverse lignocellulosic attributes contribute to the wall digestibility to obtain a high yield of fermentable sugars.

Distinct lignocellulosic attributes have been identified as key factors influencing biomass digestibility (reviewed by Hatfield et al., 2016). Various strategies have been developed to modify these plant attributes and improve the saccharification yield, including the breeding of grass varieties with altered wall structures/compositions (Bhatia et al., 2017; Yoshida et al., 2021). For example, cellulose crystallinity has been defined as a negative factor in saccharification yield of lignocellulosic biomass from maize, wheat, and rice (Wu et al., 2013; Jia et al., 2014). The structural attributes of cellulose microfibrils such as size, degree of polymerization and in particular crystallinity are important parameters determining the rigidity and degradability of grass wall polymers (Liu et al., 2013; Zhang et al., 2020). For example, the crystallinity of cellulose fibrils limits the accessibility of hydrolytic enzymes (i.e., cellulases) due to a dense crystal structure. Strategies to reduce cellulose crystallinity to improve saccharification efficiency in plants had only limited success as it severely restricts plant growth and development leading to a low plant biomass yield (Harris et al., 2012; Zhang et al., 2020).

Instead, modification of hemicelluloses such as GAX or MLG to increase wall digestibility has been proposed as a suitable alternative. Altering both GAX backbone and side-chain substituents affect the polymer conformation, impacting the interaction with other wall components such as cellulose and lignin and thus influencing the wall recalcitrance to degradation (Busse-Wicher et al., 2014; Pereira et al., 2017). For example, glucose and xylose yields released during saccharification are increased in plants with reduced xylan glucuronosylation (Lyczakowski et al., 2017). Although the study was performed in Arabidopsis and white spruce, this strategy could also increase the saccharification yield in grass species, where GAX is highly substituted with glucuronic acid.

Similarly, reducing the degree of xylan *O*-acetylation by knocking down genes involved in this modification results in increased saccharification yield in multiple plant species such as rice, Arabidopsis, or poplar (Xiong et al., 2013; Pawar et al., 2017; Zhang et al., 2017). But *O*-acetylation seems necessary for the establishment of a certain pattern of xylan decorations that enable a conformation compatible with the docking of xylan molecules onto the hydrophilic face of cellulose microfibrils (Grantham et al., 2017). This xylan-cellulose interaction is essential for development of normal secondary cell walls, so xylan hypoacetylation mutants often display severe growth defects (Manabe et al., 2013; Xiong et al., 2013; Schultink et al., 2015; Ramírez et al., 2018; Ramírez and Pauly, 2019). Alternative strategies aimed at reducing GAX arabinosyl-substituents by overexpression of an arabinofuranosidase (*OsARAF*) result in a ~46%–70% improvement in saccharification rate correlated with a decrease in arabinose content by ~20%–25% (Sumiyoshi et al., 2013). Likewise, knocking out a group of UDP-xylose epimerases (*UXE*) and xylan arabinosyl-transferase (*XAT*) genes involved in arabinose side chain synthesis also decreased xylan arabinose content and improved saccharification yield of rice straw (Chen et al., 2021). Interestingly, *uxe* and *xat* rice mutants or *OsARAF* overexpression plants have no visible grow defects despite the potential structural alterations due to changes in cellulose-xylan interactions.

Increasing the MLG content as a strategy to improve the saccharification yield in grass species has also been successfully utilized. For example, overexpression of an MLG synthase (*cellulose synthase-like F 6*) in grasses results in a high MLG content and a concomitant increase in saccharification yield. However, the biotechnological application of this strategy has some limitations, as severe associated developmental defects have been reported for example in barley and *Brachypodium dystachlon* (Burton et al., 2011; Vega-Sanchez et al., 2015; Kim et al., 2018). An alternative approach with no detrimental effects on plant yield and performance is the manipulation of MLG degradation instead (Kraemer et al., 2021; Fan et al., 2022). Due to its relatively simple structure MLG is easily degraded by hydrolytic enzymes. In fact, MLG is endogenously turned over by grass species during the night likely to obtain glucose under energy-limiting conditions. Mutations in the MLG hydrolase 1 (MLGH1), the enzyme responsible for the dark-induced degradation of MLG, result in higher accumulation of this hemicellulosic polymer accompanied by a ~30% increase in saccharification yield in maize (Kraemer et al., 2021).

In addition to altering content and composition of cell wall polysaccharides, manipulation of lignin has also been used to reduce lignocellulosic recalcitrance (Chen and Dixon, 2007; Fu et al., 2011; Mottiar et al., 2016; Halpin, 2019). A series of genes involved in the synthesis and metabolism of lignin have been identified and their functions characterized i.e. *PAL*, *4CL*, *C4H*, *CCR*, *CAD*, *HCT*, *C3H*, *CSE*, *CCoAOMT*, *F5H*, and *COMT* (reviewed by Vanholme et al., 2019 and Dixon and Barros, 2019). The down-regulation of these genes alters the lignin content and/or composition reducing lignin recalcitrance and significantly improving the saccharification efficiency of a variety of plant tissues and species (Wang et al., 2015; Halpin, 2019). However, strong lignin modifications in plants are often associated with growth defects caused by collapsed xylem vessels leading plant lodging and dwarfism (Voelker et al., 2011). Alternative approaches have been proposed, aiming to fine-tune lignin modifications to increase saccharification without impacting plant growth and development based on the spatial and temporal characteristics of lignin at both tissue and cellular level (Wang et al., 2022). Lignin-deficient mutants often display a brown midrib (bm) phenotype characterized for a brownish-red coloration of the leaf midribs and stems (reviewed by Liu et al., 2018). For example, *bm1* and *bm3* mutants affected monolignol biosynthetic genes show diverse defects in lignin content and composition in maize, sorghum, or ryegrass associated with enhanced digestibility without significant negative effects on either plant fitness or biomass production (Vignols et al., 1995; Halpin et al., 1998; Tu et al., 2010; Li et al., 2015).

Mechanical, chemical, and/or physical pre-treatments can also be used to increase the digestibility of plant lignocellulosic material (reviewed by Galbe and Wallberg, 2019). These pre-treatments aim at altering the wall architecture, removing major inhibitory barriers to wall degradation for example reducing the crystallinity and degree of polymerization of cellulose, increasing the polymer surface area available for the hydrolytic enzymes, or enhancing the susceptibility to hydrolysis of the carbohydrate substrates (reviewed by Ashokkumar et al., 2022). Some of the common strategies include pretreatments with acid/alkaline solutions, organic solvents, steam explosion or compressed hot water.

In the present study, we describe a large-scale forward genetic approach resulting in the identification of a collection of chemically mutagenized maize (*Zea mays* L.) mutants altered in their lignocellulosic composition and/or saccharification yield with no obvious developmental defects. The identified *candy-leaf* (*cal)* mutants are a valuable tool not only to understand recalcitrance of grass lignocellulosic biomass to enzymatic deconstruction but also to decipher grass-specific aspects of cell wall biology.

## Results

### Identification of *candy-leaf* mutants

A forward genetic screen was designed to identify maize plants with differences in the cell wall composition and/or properties. For that purpose, ethyl methanesulfonate (EMS)-mutagenized pollen was used to fertilize A619 ears (Neuffer, 1982; Lewis et al., 2014). M1 plants were then self-pollinated and the resulting M2 screened for wall structural alterations. To maximize the probability to identify independent mutations, M2 progenies obtained from singular M1 plants were screened individually. The focus of the analytical screen were three assays (chemotypes) performed on isolated maize wall preparations from the second leaf of 2-week-old maize seedlings. As developmental mutants might show secondary defects in the cell wall composition/content, individuals exhibiting dwarfism, chlorosis or similar macroscopic defects were excluded. Initially, wall materials were treated with a mild acid. Under these conditions, matrix carbohydrates are hydrolyzed into their constituent monosaccharides, while crystalline cellulose remains mostly intact (Foster et al., 2010b). We used this method as a proxy to identify differences in hemicellulose composition. Then, we determined the amount of wall-bound acetate (Rosa et al., 2017; Ramírez et al., 2018). Many hemicellulosic polysaccharides can be heavily *O*-acetylated *via* ester bonds modifying the physiochemical properties of the polymers altering not only the interaction but also their enzymatic hydrolysis (Pauly and Ramirez, 2018). Last, we determined the saccharification yield by measuring the amount of glucose released by hydrolysis using a commercial enzyme cocktail (Accellerase 1500^R^). Using this pipeline nearly 12,000 M2 individuals were screened revealing 51 candidate outliers showing a ≥ 20% difference in one or more of the chemotypic values (**Table 1**; **Supplementary Table 1**). These candidates were self-pollinated and rescreened in the next generation for the presence of their respective chemotype(s). For 14 candidates, the chemotype(s) could be confirmed in the subsequent generation and were therefore deemed heritable. The remaining 37 were initially discarded due to several reasons. Most of them (21/37) died before flowering or showed developmental abnormalities in adult plants, including severe dwarfism, chlorosis or sterility. From the rest (16/37), their chemotype could not be reproduced in the descendants and might thus correspond to false positives or non-heritable traits. The 14 confirmed candidates were back-crossed to the A619 original inbred in order to segregate spurious mutations and/or crossed to the B73 reference line for mapping purposes. After backcrossing, 8 *cal* mutants showed segregations consistent with monogenic traits, 7 being recessive (*cal1*, *cal2*, *cal3*, *cal4*, *cal5*, *cal7* and *cal8)* and one dominant (*cal6*). The remaining 6 showed abnormal segregations indicating complex genetic causes. Among the *cal* collection, two mutants - *cal1* and *cal6* - showed an increased saccharification yield (**Table 1**). While *cal1* exhibited an increase in Glc content, in *cal6* Ara and Xyl content was increased. Five other mutants showed reduced saccharification yields. Four of them - *cal3*, *cal4*, *cal5* and *cal8* - did not show major differences in the other assays, while *cal3* additionally showed a decrease in its wall Glc content. Finally, *cal7* showed an increase in Ara content (**Table 1**). We next focused on the characterization of these 8 *cal* mutants exhibiting clear monogenic inheritances. The subsequent analyses were performed in *cal1* and *cal2* crossed twice to B73, and *cal3* to *cal8* backcrossed once to A619.

**Table 1:** Summary *cal* mutant screen.

ID	Ac	Glc	Xyl	Ara	Sacch	# germ	# chemo	Chemotype	M3	BC segregation	Mutant
69-3	nd	73	118	135	nd	11	1	High Ara, Low Glc	positive	undetermined	
107-7	nd	100	79	92	nd	12	2	Low Xyl	positive	undetermined	
**236-1**	**nd**	**386**	**95**	**99**	**nd**	**12**	**3**	**High Glc**	**positive**	**recessive**	***cal1***
330-3	98	132	108	99	110	12	5	High Glc	positive	undetermined	
**383-10**	**102**	**93**	**99**	**100**	**68**	**11**	**4**	**Low Sacch**	**positive**	**recessive**	***cal2***
384-4	100	103	101	100	132	9	2	High Sacch	positive	undetermined	
388-3	98	94	97	127	81	10	7	High Ara	positive	undetermined	
**499-1**	**98**	**89**	**92**	**90**	**72**	**9**	**1**	**Low Saccharification**	**positive**	**recessive**	***cal5***
**582-2**	**99**	**110**	**99**	**126**	**104**	**12**	**2**	**High Ara**	**positive**	**recessive**	***cal7***
**590-3**	**105**	**110**	**122**	**124**	**125**	**9**	**4**	**High Sacch, High Xyl, High Ara**	**positive**	**dominant**	***cal6***
**613-2**	**89**	**nd**	**nd**	**nd**	**74**	**11**	**1**	**Low Sacch**	**positive**	**recessive**	***cal4***
614-4	94	75	112	114	70	11	2	Low Sacch	positive	undetermined	
**631-8**	**92**	**68**	**82**	**87**	**62**	**10**	**2**	**Low Sacch, Low Glc**	**positive**	**recessive**	***cal3***
**736-8**	**94**	**76**	**93**	**96**	**50**	**8**	**1**	**Low Sacch**	**positive**	**recessive**	***cal8***

Mutant outliers whose chemotype was confirmed in the M3. Highlighted in bold are the cal mutants with a monogenic segregation. Wall-bound acetate content (Ac), Saccharification (Sacch) and relative monosaccharide content are shown as percentage of the average value of the screened individuals. Number of germinated plants per M2 (#germ) and number of individuals showing the corresponding chemotype (#chemo) are also indicated. The M3 column indicates, if the chemotype was observed in the progeny after self-crossing of the outlier. Backcross (BC) segregation indicates if the mutant segregation is compatible with a dominant, recessive, or undetermined trait. Mutant column shows the assigned cal name. Full table with all outliers identified in the screen is shown in **Supplementary Table 1**.

### Maize *candy-leaf* mutants show altered saccharification yield

**Figure 1:**
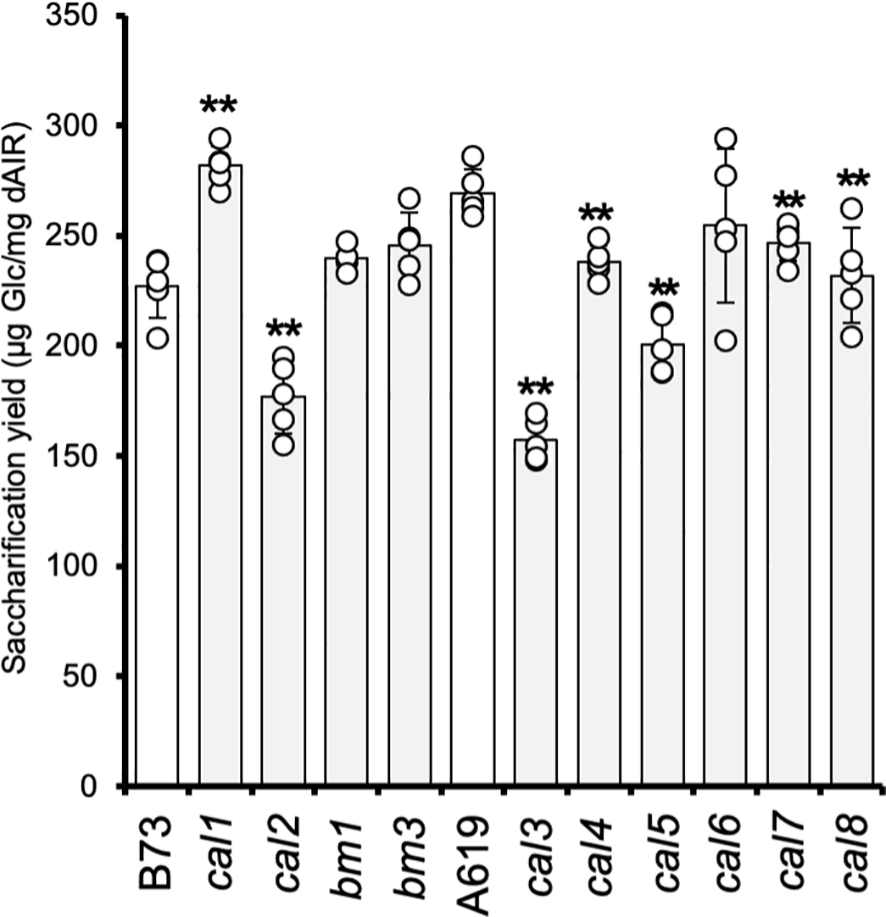
Saccharification of *cal* mutant seedlings. The values correspond to the mean ± SD (n = 5). *cal1*, *cal2*, *bm1*, and *bm3* are in the B73 genetic background. *cal3*, *cal4*, *cal5*, *cal6*, *cal7*, and *cal8* are in the A619 genetic background. dAIR destarched alcohol insoluble residue. Asterisks indicate levels of statistical significance between each mutant and corresponding wild-type plant determined by two-tailed unpaired Student *t*- test at *p*-value < 0.01 (**).

To compare the cell wall digestibility of the newly identified *cal* mutants, we analyzed the glucose yields released by enzymatic saccharification from walls isolated from 2-week-old seedlings (**Figure 1**). Lignin is generally negatively correlated with wall digestibility (Méchin et al., 2000; Chen and Dixon, 2007; van der Weijde et al., 2015). It has been reported that maize *brown-midrib* (*bm*) mutants with reduced lignin content and/or alteration in the lignin composition enhance the wall digestibility by enzymatic saccharification (Sattler et al., 2009; Christensen and Rasmussen, 2019). Hence, both of these *bm* mutants, i.e., *bm1* and *bm3*, were included in this study and backcrossed three times to B73. The previously described *cal1* mutant was also included for comparison (Kraemer et al., 2021). *cal1* seedlings showed a 24% increase in saccharification yield compared to the corresponding B73 wild-type control. In contrast, the *cal2* mutant - also in B73 genetic background - exhibited a significant decrease in the saccharification yield with a ~22% reduction compared to the control. The results showed that *bm1* and *bm3* did not show a significant increment in their saccharification yields, probably due to the low tissue lignification at the seedling stage selected for this analysis. The *cal3*, *cal4*, *cal5*, and *cal8* mutants – in the A619 genetic background - showed significant reductions in their saccharification yields confirming the results found in the screen (**Figure 1**). The lowest yield among those mutants was observed in *cal3* with a ~42% reduction. For *cal4*, *cal5* and *cal8*, the observed saccharification yields decreased by 12%, 26%, and 14%, respectively. Interestingly, the *cal7* mutant, initially selected in the screen based on increased Ara content, also showed a significant decrease in yield (8%). In the case of the *cal6* mutant, also in A619 background, the results were inconclusive (**Figure 1**). Despite some *cal6* plants exhibit an increase in the saccharification yield consistent with the results obtained in the mutant screen, a high variability among the different individuals analyzed was observed. This result might suggest genetic heterogeneity in the dominant *cal6* mutant, although incomplete penetrance cannot be excluded.

### Altered saccharification in *cal* mutants is associated with differences in cell wall composition

To further investigate the existence of wall changes underlying the alteration in saccharification observed in the different *cal* mutants, we performed a more detailed cell wall compositional analysis. Crystalline cellulose content, monosaccharide composition, lignin content, and acetate content were determined from the same isolated wall materials used for the saccharification yield assay. To expand the characterization of the *cal* mutant collection, the method of determining the absolute and relative monosaccharide composition was adjusted. Here, a streamlined one-step/two-step method using different concentrations of sulfuric acid was employed to hydrolyze paired destarched alcohol insoluble residue (dAIR) samples to simultaneously quantify crystalline cellulose content and matrix sugar composition (Yeats et al., 2016; Menna et al., 2020). With this method, the main chemotypes identified in the screen could be confirmed for all *cal* mutants analyzed, but also some other differences became apparent (**Table 2**; **Supplementary Table 2**). The *cal1* mutant has been previously shown to accumulate high amounts of MLG. Accordingly, an increase in the relative content of matrix Glc was observed with a concomitant reduction in the relative abundance of the rest of the matrix monosaccharides, i.e., Ara, Gal and Xyl. The other lignocellulosic attributes remained unaltered compared to the wildtype.

**Table 2:** Cell wall monosaccharide composition of cal mutant maize seedlings.

Genotype	Arabinose	Galactose	Glucose	Xylose	Cellulose	Lignin	Acetate
**B73**	18.1 ± 0.6	4.9 ± 0.2	38.0 ± 1.3	39.0 ± 0.9	291.8 ± 15.7	68.1 ± 8.6	13.7 ± 0.3
***cal1***	15.0 ± 0.6**	3.8 ± 0.1**	46.8 ± 1.1**	34.4 ± 0.8**	275.0 ± 19.9	74.9 ± 10.8	13.2 ± 0.7
***cal2***	24.0 ± 2.4**	4.9 ± 0.3	24.2 ± 7.2**	46.9 ± 4.6**	256.6 ± 9.3**	79.9 ± 11.2	17.5 ± 1.1**
***bm1***	17.7 ± 0.4	4.4 ± 0.2**	35.7 ± 1.6*	42.2 ± 1.5**	309.3 ± 16.5	72.7 ± 13.0	13.9 ± 0.7
***bm3***	23.7 ± 0.7**	6.2 ± 0.3**	24.1 ± 2.2**	46.0 ± 1.7**	328.2 ± 27.5*	74.0 ± 8.7	17.3 ± 1.4**
**A619**	19.5 ± 0.9	4.5 ± 0.2	38.0 ± 2.7	38.1 ± 1.6	285.0 ± 8.9	71.1 ± 7.8	17.1 ± 1.4
***cal3***	26.7 ± 1.3**	6.0 ± 0.2**	16.1 ± 2.7**	51.2 ± 1.4**	242.5 ± 10.9**	76.9 ± 8.4	16.9 ± 1.7
***cal4***	23.6 ± 0.9**	5.2 ± 0.1**	26.3 ± 2.9**	44.8 ± 2.0**	273.9 ± 8.4	73.7 ± 7.8	16.5 ± 1.7
***cal5***	27.1 ± 1.0**	5.9 ± 0.3**	17.6 ± 3.3**	49.4 ± 2.3**	278.1 ± 7.8	65.5 ± 5.2	17.0 ± 1.2
***cal6***	19.4 ± 1.2	4.2 ± 0.3	35.3 ± 3.3	41.1 ± 2.3**	325.4 ± 34.2*	68.1 ± 6.1	15.5 ± 0.8
***cal7***	22.4 ± 0.8**	4.9 ± 0.1**	28.3 ± 1.7**	44.3 ± 0.9**	303.6 ± 10.6*	66.7 ± 5.9	16.5 ± 0.8
***cal8***	23.0 ± 0.9**	5.1 ± 0.4*	26.8 ± 2.4**	45.0 ± 1.4**	301.7 ± 14.4	78.8 ± 3.4	16.4 ± 0.8

Monosaccharide composition values are shown as the mean and average of the relative content of each monosaccharide (in %) of 5 biological replicates. 100% corresponds to the sum of Ara, Gal, Glc and Xyl. cal1, cal2, bm1, and bm3 are in B73 genetic background. cal3, cal4, cal5, cal6, cal7, and cal8 are in A619 genetic background. Asterisk(s) indicate levels of statistical significance between each mutant and corresponding wild-type plant determined by two-tailed unpaired Student t-test at p-value < 0.01 (**), p-value < 0.05 (*).

In *cal2*, exhibiting a low saccharification, a 36% reduction in the matrix Glc content is observed with this method, in contrast to what was found in the screen (no difference). In addition, a 22% decrease in the crystalline cellulose content was detected. Also noteworthy is a 22% increase in wall-bound acetate and an increase in the relative abundances of matrix Ara and Xyl but not Gal indicating a more complex chemotype with more than one wall component, polymer interactions, and/or wall architecture altered (**Table 2**). The *cal3*, *cal4*, *cal7* and *cal8* mutants, all showing a reduced saccharification yield, also show significant decreases in the relative matrix Glc content, in particular the 57% and 53% reductions in *cal3* and *cal5*, respectively. In all of these mutants, the relative abundances of the remaining matrix monosaccharides increase accordingly (**Table 2**). However, when absolute amounts are computed other differences can be found (**Supplementary Table 2**). *cal3* showed a 15% reduction in the crystalline cellulose content compared to the wildtype control. As for *cal6*, a conspicuous increase in crystalline cellulose and an increase in the relative Xyl content were detected, although the variability among the different individuals analyzed was exceptionally high, similar to what was previously observed in the case of the saccharification yield measurements (**Table 2**). Together, these results demonstrate that the alteration in saccharification yield in young leaves of the *cal* mutants are associated with changes in cell wall carbohydrate content and/or composition.

### Saccharification and cell wall composition of mature tissues of *cal* mutants

Among the *cal* collection, we selected the *cal1*, *cal2*, and *cal3* mutants to further investigate their lignocellulosic attributes in adult leaves and stalks as these mutants exhibited some interesting differences in terms of saccharification yield correlated with cell wall structural defects at the seedling stage. Adult plants of *cal1*, *cal2* and *cal3* mutants showed no obvious differences compared to wide-type in terms of plant height or architecture (**Supplementary Figure 1A**). In addition, none of these *cal* mutants display a change in the midrib coloration as the *bm1* and *bm3* mutants. (**Supplementary Figure 1B**).

**Figure 2:**
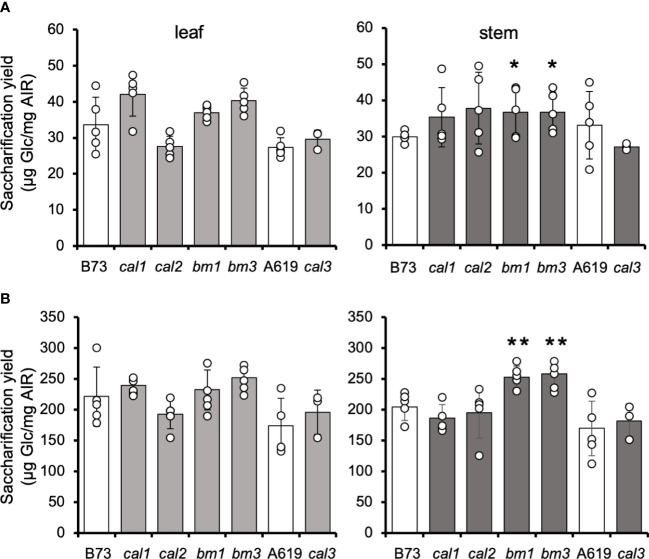
Saccharification yield of cal adult tissues. Amounts of glucose released from dAIR after 20-h enzymatic digestion from untreated **(A)** and NaOH-pretreated **(B)** mature leaves and stems. Values are means ± SD (n ≥ 3. Asterisks indicate significant differences from the wild type plants using the unpaired Student's t-test *p*-value < 0.05 (*); *p*-value < 0.01 (**).

In contrast to the results obtained in seedlings, no differences were found in the saccharification yield of adult leaves and stalks derived from the *cal1*, *cal2*, and *cal3* mutants (**Figure 2A**). However, the *bm1* and *bm3* controls showed an increase in saccharification efficiency in heavily lignified tissues, i.e., stems, but not leaves (**Figure 2A**).

The saccharification performance of *cal* and *bm* mutant stem tissue was also assessed after alkali pretreatment (**Figure 2B**). The pretreatment of lignocellulosic biomass with 25 mM NaOH increased the saccharification yield of all tissues by 6-fold. However, no differences were found in *cal1*, *cal2*, or *cal3* compared to the respective wildtype controls. Conversely, pretreatment of *bm1* and *bm3* stem tissue increased the saccharification yield by 24% similar to the data published previously (Halpin et al., 1998; Marita et al., 2003; Barrière et al., 2013; Xiong et al., 2020).

Besides the saccharification efficiency, we also analyzed the wall composition of the mature senesced leaves and stems from the selected *cal* and *bm* mutants and corresponding wide-type plants (**Table 3**; **Supplementary Table 3**). In general, the main differences observed in *cal1*, *cal2*, and *cal3* mutant seedlings were reduced if not absent in adult tissues. This might suggest that the affected genes function primarily in processes and/or tissues associated with the seedling development. This could also explain the lack of differences in the saccharification yield of adult tissues. However, a 27% increase in matrix Glc was detected in *cal1* stems consistent with the overaccumulation of MLG as also reported previously in this mutant (Kraemer et al., 2021). In *cal2*, the decrease in relative Glc content was lower than in seedling tissue (18% versus 36.3%) and only significant in leaves but not stems (**Table 2**, **3**). An increase in the relative Ara content was observed in adult leaves (+17%) and stems (+50%), similar to what was observed in seedlings. In *cal3* adult plants a 21% increase in relative Ara content and a 23% decrease in relative Glc abundance was observed in the stem tissue. The remaining lignocellulosic attributes remained unaltered. Under the growth conditions used here, only *bm3* but not *bm1* mutant stems showed a decrease in lignin content compared to the B73 wild-type control. In fact, the lignin content in *bm1* stems was even slightly increased. The lignin content has been reported in diverse *bm1* alleles ranging from 4-20% reduction to no differences depending on the study and the method used (Halpin et al., 1998; Marita et al., 2003; Barrière et al., 2013; Xiong et al., 2020). A comparative study also of the monolignol composition is needed to determine the nature of this chemotypic variation.

**Table 3:** Cell wall composition of *cal* mutant mature tissues.

Genotype	Mature tissue	Arabinose	Galactose	Glucose	Xylose	Cellulose	Lignin
**B73**	leaf	15.1 ± 1.1	6.6 ± 0.9	15.4 ± 2.2	63.0 ± 2.8	299.0 ± 66.5	184.8 ± 10.8
***cal1***	leaf	16.1 ± 1.0	7.1 ± 0.5	16.2 ± 1.3	60.6 ± 0.7	280.7 ± 18.8	179.8 ± 9.6
***cal2***	leaf	17.7 ± 0.8**	7.1 ± 0.4	12.6 ± 1.2*	62.5 ± 1.5	239.4 ± 26.6	190.4 ± 7.1
***bm1***	leaf	16.2 ± 1.5	6.9 ± 0.4	14.2 ± 1.5	62.7 ± 0.6	297.2 ± 53.7	179.9 ± 26.3
***bm3***	leaf	16.5 ± 0.7*	7.1 ± 0.3	12.9 ± 0.9*	63.6 ± 0.8	293.0 ± 24.0	184.3 ± 5.8
**A619**	leaf	16.8 ± 0.5	6.6 ± 0.7	10.7 ± 1.3	65.9 ± 2.4	251.4 ± 31.4	170.0 ± 31.1
***cal3***	leaf	17.8 ± 1.5	6.9 ± 0.6	10.3 ± 0.8	64.9 ± 1.4	261.8 ± 36.9	179.5 ± 25.0
**B73**	stem	11.6 ± 1.0	3.6 ± 0.5	8.7 ± 0.6	76.1 ± 1.4	319.1 ± 22.0	179.8 ± 4.7
***cal1***	stem	12.5 ± 0.7	3.6 ± 0.5	10.6 ± 1.4*	73.2 ± 2.0*	319.1 ± 35.3	187.0 ± 8.5
***cal2***	stem	17.6 ± 1.6**	4.0 ± 0.4	7.9 ± 0.9	70.5 ± 1.3**	298.9 ± 14.2	168.4 ± 11.0
***bm1***	stem	12.0 ± 0.8	3.6 ± 0.3	8.6 ± 0.8	75.9 ± 0.4	344.9 ± 23.0	200.2 ± 6.0**
***bm3***	stem	12.2 ± 1.1	3.4 ± 0.4	7.1 ± 0.7**	77.3 ± 2.1	354.3 ± 32.2	159.6 ± 12.5**
**A619**	stem	16.5 ± 0.5	4.4 ± 0.3	7.6 ± 0.8	71.6 ± 1.4	337.2 ± 33.4	151.5 ± 19.8
***cal3***	stem	20.0 ± 0.3**	4.9 ± 0.1	5.8 ± 0.2**	69.3 ± 0.5*	280.8 ± 49.9	174.9 ± 6.3

Monosaccharide composition values are shown as the mean and average of the relative content (in %) of each monosaccharide (n ≥ 3). 100% corresponds to the sum of Ara, Gal, Glc and Xyl. Crystalline cellulose and lignin content are shown in μg mg-1 AIR. cal1, cal2, bm1, and bm3 are in B73 genetic background. cal3 are in A619 genetic background. Asterisk(s) indicate levels of statistical significance between each mutant and corresponding wild-type plant determined by two-tailed unpaired Student t-test at p-value < 0.01 (**), p-value < 0.05 (*).

## Discussion

The composition and structure of grass cell walls differ from those of dicot species. Due to the high economic importance of some grass species including cereals such as maize, wheat, rice, or sorghum, large efforts have been made to characterize this peculiar wall type. Forward and reverse genetic approaches have been used to identify some mutants with altered cell wall structure and composition in several grass species (Vermerris et al., 2010; Hu et al., 2020; Xiong et al., 2020; Kraemer et al., 2021; Liu et al., 2021; Fan et al., 2022). These mutants have been instrumental to uncover some grass-specific aspects of the cell wall biology. However, the wall mutant repertoire in grasses is very limited in comparison to dicots such as the model system Arabidopsis. The new collection of candy-leaf maize wall mutants reported in this study aims at contributing reducing this gap and providing new tools for the grass wall research community. 8 *cal* mutants have been identified with diverse defects in hemicellulose composition and/or cellulose content at the seedling stage. All *cal* mutants also exhibit alterations in their saccharification yield confirming the importance of lignocellulosic structure for this important economical trait, particularly considering the absence of associated growth and developmental defects in the *cal*-mutants.

Despite the descriptive nature of this study, the compositional information compiled here allows us to speculate on the possible wall defects responsible for the observed differences in the saccharification yield of the *cal* mutant collection. For example, the *cal2* mutant was identified in our screen based on a reduction in saccharification yield (**Supplementary Table 1**). Further analyses showed an associated reduction in cellulose content (**Table 1**). As the largest fraction of glucose released during the saccharification of maize wall material originates from the hydrolysis of cellulose, it seems likely that a lower abundance of this polymer may be the cause of the decrease in the *cal2* saccharification yield (**Figure 1**). Similarly, the decrease in crystalline cellulose detected in the *cal3* mutant might also explain the reduced saccharification yield (**Figure 1**). Both mutants also shared other differences in the matrix composition such as an increase in the Ara content in seedlings and some mature tissues (**Table 1**, **Table 2**, **Supplementary Table 1**, **Supplementary Table 2**). In grasses Ara is present in Ara-containing oligosaccharide sidechain substituents decorating the xylan backbone. The Ara substitution pattern is believed to be essential for the conformation of GAX determining its association strength with cellulose fibrils. The reduction in Ara content in *cal2* and *cal3* may result in altered cellulose-GAX interactions influencing wall digestibility. The opposite effect has been reported in rice mutants with reduced GAX arabinosylation, showing a low Ara content associated with an increased saccharification yield of rice straw (Chen et al., 2021). Alternatively, Ara is also present in pectic polysaccharides and arabinogalactan derived glycans. Additional experiments are needed to identify if and which of these wall components are altered in *cal2* and *cal3.*

The similarities in the chemotypes associated to *cal2* and *cal3* might indicate that both mutations are allelic, i.e., the causative mutations affect the same gene. However, some differences arose during the analysis supporting the contrary. First, the two mutants seem to have different tissue-specific patterns in adult tissues regarding matrix composition. While the differences in Ara and Glc in *cal2* are found in most adult tissues, in *cal3* they are only clearly observed in stems. Second, unlike in *cal3* an increase in the total wall-bound acetate was detected in *cal2* seedlings. Third, the two methods used to determine the hemicellulose composition revealed an interesting difference between *cal2 and cal3*. While the hydrolysis of walls using diluted sulfuric acid resulted in a reduced Glc content in both mutants compared to the control, only *cal3* showed significant decreases when TFA hydrolysis was used. Although further research is needed to clarify this apparent discrepancy, one could hypothesize that the two methods have a different efficiency in hydrolyzing *cal2* walls due to a specific alteration not present in *cal3* (e.g., different cellulose crystallinity or amorphous/crystalline ratio in *cal2*).

The low saccharification yields observed in *cal4, cal5, cal7* and *cal8* mutant seedlings seem to correlate with a reduced Glc content in the hemicellulose fraction. In fact, the largest yield reductions are found in *cal3* and *cal5*, mutants with the lowest relative Glc abundances, while only mild reductions in saccharification are detected in *cal4*, *cal7* and *cal8* plants showing a less severe decrease in the hemicellulosic Glc content. The chemotype of these low-glucan *cal* mutants is inverse to *cal1* with an increased Glc content and high saccharification yields. The *cal1* mutant over-accumulates MLG due to a mutation in MLGH1 the enzyme responsible for dark-induced MLG hydrolysis (Kraemer et al., 2021). One possibility could be that the walls of these low-glucan *cal* mutants contain less MLG as a result of an exacerbated MLG degradation or reduced level of MLG synthesis. Such mutants could shed light into the mechanisms of MLG synthesis and turnover currently under debate.

The results obtained in this study validate the need for a future in-depth analysis of the *cal* mutant collection including identification of the causative mutations and the affected corresponding genes. At least 8 *cal* mutations seem to be heritable and show mendelian segregations. Furthermore, the single seed descent approach employed in this mutant screen makes it very unlikely that the same mutation is responsible for the chemotypes observed in the different *cal* mutants. Nevertheless, it is still possible that different mutations are present in the same pathway resulting in the similar chemotypes associated with some of the *cal* mutants. In conclusion, the new *candy-leaf* mutant collection expands the current repertoire of maize cell wall mutants and show great potential in contributing to addressing research questions specific to grass cell walls as well as the underlying molecular factors affecting enzymatic saccharification of grass lignocellulosic biomass.

## Materials and methods

### Plant materials and growth

For the mutant screen, plants were grown in the greenhouse under 16-h d/8-h night photoperiod, temperatures of low 20°C/high 25.6°C and watered two times per day with 100 ppm M, W, F w 20/20/20 fertilizer (Peters Professional). For detailed analyses, maize seedlings were grown for 2 weeks in a phytotron (Weiss Technik) under 16 h day, 24°C/8 h night, 20°C conditions. For cell wall analyses, seedlings were transferred to a dark room for 20 h before and the second leaf blade harvested. Senesced stems, and senesced leaf blades including sheath were harvested from plants grown in the field.

Seeds from wild-type (A619 and B73) and *bm* maize mutants and were obtained from a collection at the Plant Gene Expression Center, Agricultural Research Service, U.S. Department of Agriculture, Albany, California. In order to genotype *bm* mutants, genomic DNA was extracted using the protocol described by Lunde et al., 2018. For the *bm1* mutant, PCR was performed using the primers 5’-CATGACGACAGGACAACCAC-3’ and 5’-TTCAGCGTTATCTTGCATGC-3’ resulting in a 446 bp amplicon. PCR products were digested with *Apa*I resulting in two bands in the wildtype (259 bp and 187 bp) and no digestion in the *bm1* mutant due to a G-A mutation in the Zm00001eb234730 gene. For *bm3* genotyping, PCR was performed using the primers: 5’-CTTGTATGCGCTGATCTGATTC-3’ and

5’-GATGAGATGGCATGGCTGC-3’. In wild-type plants, the resulting amplicon has a size of 749 bp, while no amplicon is observed in *bm3* plants due to an insertion in the Zm0000eb172420 gene.

### Cell wall isolation

The mature stems and leaves (including sheath) were immediately dried after harvesting at 50°C for 3 days in a Innova^®^ 44 incubator (Eppendorf). The dried tissues were cut and ground into crude powder in a GM200 mixer mill (Retsch) with the settings: 15 s “cut interval on” at 4000 RPM followed by 15 s “cut interval off” at 10000 RPM. The seedling leaves and ground powder of mature tissues were freeze-dried at for 48 h in a Coolsafe system (Scanvac) and homogenized in 2 mL screw cap tubes containing two 5-mm steel balls for 2 min at 30 Hz in a MM400 mixer mill (Retsch). Preparation of de-starched AIR (dAIR) was performed as described in Foster et al. (2010a).

### Determination of monosaccharide composition

Two different methods were used for the quantitative analysis of monosaccharide composition. For the mutant screen, hydrolysis of dAIR with 2 M TFA at 121°C for 1h was performed according to Foster et al. (2010b). Sugar mixtures were separated on a HPAEC system (Knauer Azura) equipped with a CarboPac PA20 separator column (3 x 150 mm) and a CarboPac PA20 guard column (3 x 30 mm) using the following gradient: 21 min 2 mM NaOH, 5 min 700 mM NaOH, 10 min 2 mM NaOH, with a flow rate of 0.4mL/min. Detection was performed using a PAD detector (Antec Scientific Decade Elite).

The one step-two step method for the simultaneous determination of monosaccharide composition and crystalline cellulose was performed according to Yeats et al. (2016) with minor modifications. Briefly, 50 μg ribose were added to paired dAIR samples (1 mg) and dried at 40°C under gentle air flow for 30 minutes. One of the tubes was incubated at room temperature in the presence of 50 μL 72% (w/v) sulfuric acid and 1.4 mL of water were added after 1 h. In the second tube, 1.45 mL of 4% (w/v) sulfuric acid were added. Both paired samples were autoclaved at 121°C for 1 h in a VX-120 autoclave (Systec) and diluted 1:5 with 0.5 M NaAc pH 5.2 for neutralization. Diluted samples were injected into the HPAEC-PAD system as described above. Quantification of the resulting monosaccharides was performed by comparison with increasing concentrations of known sugars using ribose as internal standard.

### Acetate content

The determination of cell wall acetate content was performed as indicated in Ramírez et al. (2018). 0.5 M NaOH (100 µL) was added to 1 mg dAIR, and reactions were incubated at 25°C for 1 h at 250 RPM constant shaking. The reaction was neutralized through the addition of an equal volume of 1 M HCl. After centrifuging, total acetic acid content of the supernatant was quantified using the Acetic Acid Kit (Megazyme).

### Lignin content

Determination of total lignin content was performed using the Acetyl Bromide Soluble Lignin (ABSL) Assay as described in Foster et al., 2010a. Briefly, 1mg dAIR was incubated in the presence of acetyl bromide solution (25% v/v acetyl bromide in glacial acetic acid) at 50°C for 3 h. After adding 2M sodium hydroxide and 0.5 M hydroxylamine hydrochloride to a final volume of 2 mL the absorption at 280 nm was measured against a blank on a spectrophotometer. Determination of the percentage of ABSL is done with the following formula:


$$\frac{abs_{280nm}\;\times \;2ml\;\times 100\%}{Coeff\;\times 0.539cm\;\times \;weight\;\left(mg\right)}$$

Where Coeff is an empiric coefficient specific for grasses = 17.75, and 0.539 cm represents the path length of the 96-well plates used.

### Saccharification yield

For the saccharification assay 1 mg dAIR was incubated with 0.5 μL of Accelerase 1500 (Genencor) in 50 mM citrate buffer (pH 4.5) plus 0.01% NaN_3_ according to Santoro et al. (2010). One 5-mm steel ball for each sample was added and enzymatic reactions were performed for 20 h at 50°C with 250 RPM constant shaking. Released glucose was measured in a YSI 2900 biochemistry analyzer following manufacturer’s instructions. In the indicated cases, samples were pretreated with 25 mM NaOH for 45 min at 121°C, and neutralized with 2% sulfuric acid before enzymatic saccharification.

## Data availability statement

The original contributions presented in the study are included in the article/**Supplementary Material**. Further inquiries can be directed to the corresponding author.

## Author contributions

CL generated the mutant populations for the screening, grew the plants and performed the necessary crosses. VR, LL, FK, and BK analyzed the plants and selected candidate outliers during the mutant screen. SW analyzed in detail the lignocellulosic attributes of the *cal* mutants. MS participated in the characterization of cal3 and cal6. SR performed the saccharification with and without base pretreatment of adult tissues. SW, MP and VR wrote the manuscript. MP and VR conceived and designed the experiments. All authors contributed to the article and approved the submitted version.
