# Supplementary figures and images for: A large-scale forward genetic screen for maize mutants with altered lignocellulosic properties
Source: Front Plant Sci. 2023 Mar 7;14:1099009. doi: 10.3389/fpls.2023.1099009 (PMC10028098; doi:10.3389/fpls.2023.1099009)

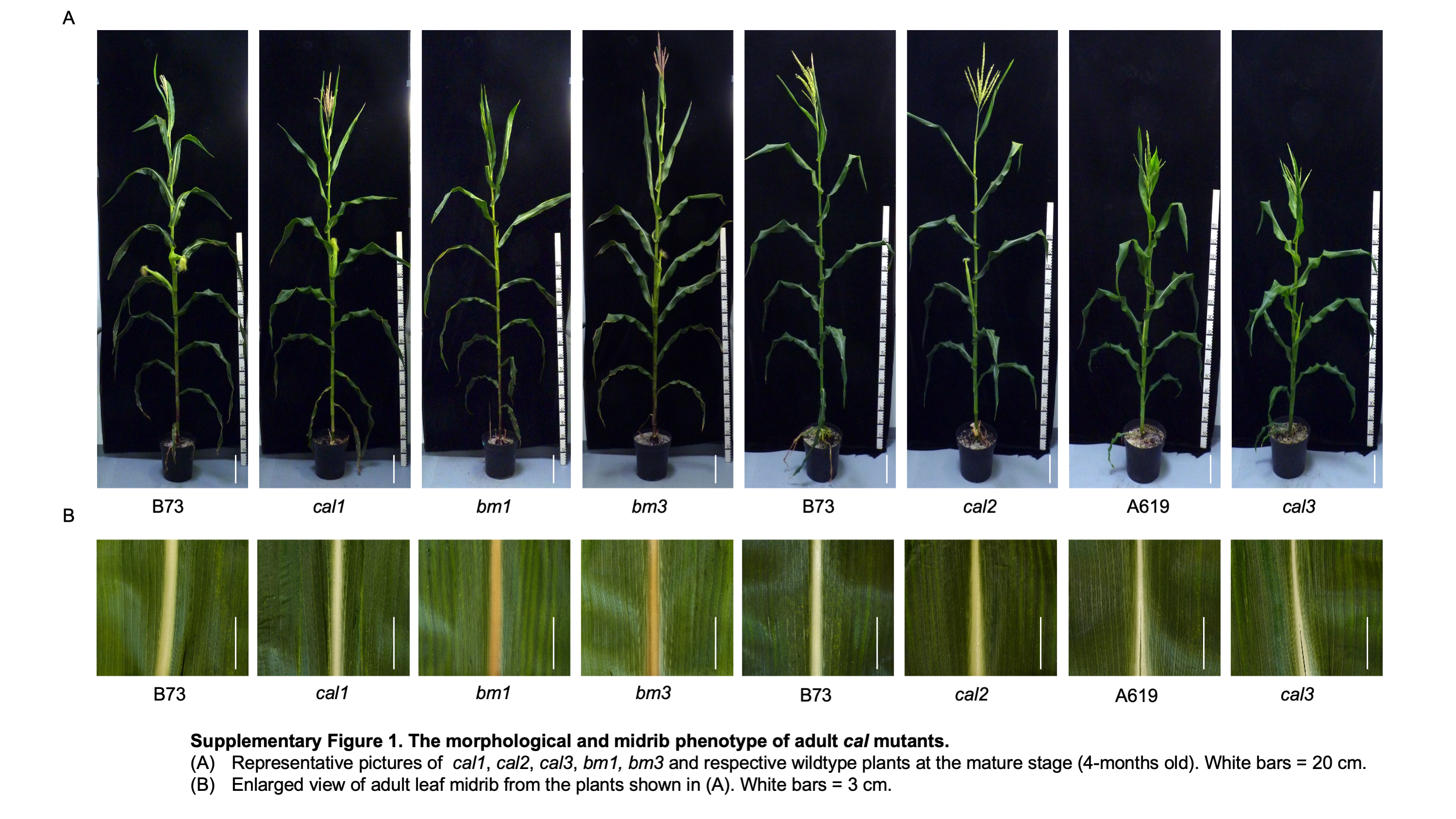

Supplement: Supplementary file 1 [file Image_1.tiff]
