# Supplementary material for: A large-scale forward genetic screen for maize mutants with altered lignocellulosic properties
Source: Front Plant Sci. 2023 Mar 7;14:1099009. doi: 10.3389/fpls.2023.1099009 (PMC10028098; doi:10.3389/fpls.2023.1099009)
Supplement: Supplementary file 2 [file Table_1.docx]

|  | **Ac** | **Glc** | **Xyl** | **Ara** | **Sacch** | **# germ** | **# chemotype** | **Chemotype** | **M3** | **BC segregation** | **Mutant** |
| --- | --- | --- | --- | --- | --- | --- | --- | --- | --- | --- | --- |
| 13-7 | nd | 201 | 98 | 99 | nd | 9 | 1 | High Glc |  |  |  |
| 69-3 | nd | 73 | 118 | 135 | nd | 11 | 1 | High Ara, Low Glc | positive | undetermined |  |
| 107-7 | nd | 100 | 79 | 92 | nd | 12 | 2 | Low Xyl | positive | undetermined |  |
| 171-6 | nd | 118 | 71 | 69 | nd | 10 | 1 | Low Ara, Low Xyl |  |  |  |
| 199-7 | nd | 105 | 68 | 62 | nd | 11 | 1 | Low Ara, Low Xyl |  |  |  |
| 222-7 | nd | 170 | 102 | 104 | nd | 9 | 1 | High Glc |  |  |  |
| **236-1** | **nd** | **386** | **95** | **99** | **nd** | **12** | **3** | **High Glc** | **positive** | **recessive** | ***cal1*** |
| 250-1 | nd | 68 | 145 | 146 | nd | 5 | 1 | Low Glc, HighXyl, High Ara |  |  |  |
| 261-4 | nd | 100 | 129 | 154 | nd | 7 | 1 | High Ara, High Xyl |  |  |  |
| 276-2 | nd | 122 | 112 | 111 | nd | 10 | 1 | High Glc |  |  |  |
| 301-9 | nd | 136 | 130 | 132 | nd | 5 | 1 | High Hemicellulose | NC |  |  |
| 314-2 | 145 | 121 | 102 | 106 | 103 | 9 | 1 | High Ac, High Glc | NC |  |  |
| 315-5 | 88 | 110 | 90 | 92 | 166 | 9 | 2 | High Saccharification | NC |  |  |
| 330-3 | 98 | 132 | 108 | 99 | 110 | 12 | 5 | High Glc | positive | undetermined |  |
| 335-4 | 92 | 117 | 86 | 118 | 137 | 7 | 1 | High Sacch | NC |  |  |
| 365-8 | 98 | 164 | 95 | 97 | 134 | 8 | 2 | High Glc, High Sacch | NC |  |  |
| **383-10** | **102** | **93** | **99** | **100** | **68** | **11** | **4** | **Low Sacch** | **positive** | **recessive** | ***cal2*** |
| 384-4 | 100 | 103 | 101 | 100 | 132 | 9 | 2 | High Sacch | positive | undetermined |  |
| 388-3 | 98 | 94 | 97 | 127 | 81 | 10 | 7 | High Ara | positive | undetermined |  |
| 409-5 | 93 | 97 | 102 | 99 | 136 | 10 | 1 | High Sacch | NC |  |  |
| 425-12 | 148 | 98 | 103 | 104 | 128 | 12 | 1 | High Ac, High Sacch | NC |  |  |
| 433-1 | 90 | 109 | 104 | 134 | 106 | 11 | 2 | High Sacch | NC |  |  |
| 479-9 | 97 | 100 | 76 | 71 | 96 | 11 | 2 | Low Xyl, Low Ara |  |  |  |
| **499-1** | **98** | **89** | **92** | **90** | **72** | **9** | **1** | **Low Saccharification** | **positive** | **recessive** | ***cal5*** |
| 528-8 | 89 | 143 | 102 | 115 | 141 | 10 | 1 | High Glc, High Sacch | NC |  |  |
| 553-10 | 154 | 134 | 109 | 122 | 100 | 10 | 2 | High Ac, High Glc, High Ara |  |  |  |
| 559-3 | 129 | 103 | 101 | 100 | 99 | 10 | 2 | High Ac | NC |  |  |
| 563-1 | 98 | 248 | 104 | 97 | 121 | 5 | 3 | High Glc, High Sacch | NC |  |  |
| 568-2 | 61 | 117 | 102 | 106 | 112 | 6 | 2 | Low Ac | NC |  |  |
| **582-2** | **99** | **110** | **99** | **126** | **104** | **12** | **2** | **High Ara** | **positive** | **recessive** | ***cal7*** |
| 588-6 | 113 | 143 | 107 | 108 | 103 | 11 | 2 | High Glc | NC |  |  |
| **590-3** | **105** | **110** | **122** | **124** | **125** | **9** | **4** | **High Sacch, High Xyl, High Ara** | **positive** | **dominant** | ***cal6*** |
| 592-2 | 70 | 95 | 92 | 93 | 100 | 11 | 2 | Low Ac | NC |  |  |
| 600-1 | 104 | 101 | 107 | 113 | 136 | 7 | 1 | High Sacch | NC |  |  |
| 601-5 | 108 | 135 | 108 | 109 | 137 | 9 | 1 | High Glc, High Sacch | NC |  |  |
| **613-2** | **89** | **nd** | **nd** | **nd** | **74** | **11** | **1** | **Low Sacch** | **positive** | **recessive** | ***cal4*** |
| 614-4 | 94 | 75 | 112 | 114 | 70 | 11 | 2 | Low Sacch | positive | undetermined |  |
| 616-4 | 97 | 99 | 116 | 119 | 131 | 7 | 2 | High Sacch | positive | undetermined |  |
| 618-2 | 96 | 55 | 80 | 82 | 58 | 6 | 1 | Low Sacch, Low Glc | NC |  |  |
| 627-9 | 151 | 51 | 54 | 50 | 90 | 11 | 1 | High Ac | NC |  |  |
| **631-8** | **92** | **68** | **82** | **87** | **62** | **10** | **2** | **Low Sacch, Low Glc** | **positive** | **recessive** | ***cal3*** |
| 635-10 | 123 | 260 | 142 | 145 | 160 | 12 | 2 | High Glc, High Sacch |  |  |  |
| 643-3 | 111 | 95 | 108 | 112 | 134 | 11 | 2 | High Sacch | NC |  |  |
| 664-7 | 95 | 91 | 93 | 90 | 79 | 9 | 1 | Low Sacch | NC |  |  |
| 672-2 | 131 | 111 | 127 | 115 | 104 | 4 | 1 | High Ac, High Xyl | NC |  |  |
| 679-3 | 115 | 175 | 121 | 128 | 110 | 8 | 1 | High Glc, High Xyl, High Sacch | NC |  |  |
| 721-1 | 86 | 135 | 129 | 132 | 101 | 7 | 1 | High Hemicellulose | NC |  |  |
| 725-1 | 98 | 96 | 90 | 85 | 131 | 4 | 1 | High Sacch | NC |  |  |
| 726-4 | 102 | 102 | 99 | 102 | 131 | 7 | 1 | High Sacch | NC |  |  |
| **736-8** | **94** | **76** | **93** | **96** | **50** | **8** | **1** | **Low Sacch** | **positive** | **recessive** | ***cal8*** |
| 740-1 | 88 | 51 | 52 | 57 | 87 | 9 | 1 | Low hemicellulose | NC |  |  |

**Supplementary Table 1. All outliers found in the *cal* mutant screen**. Highlighted in bold are the *cal* mutants with a monogenic segregation. Wall-bound acetate content (Ac), saccharification (Sacch), and relative monosaccharide content are shown as percentage of the average value of the screened individuals. Number of germinated plants per M2 (#germ) and number of individuals showing the corresponding chemotype (#chemo) are also indicated. The M3 column indicates if the chemotype was observed (positive) or not (NC) in the progeny after self-crossing the outlier. BC segregation indicates if the mutant segregation is compatible with a dominant, recessive or undetermined trait. Mutant column shows the assigned *cal* name.
