# Supplementary material for: A large-scale forward genetic screen for maize mutants with altered lignocellulosic properties
Source: Front Plant Sci. 2023 Mar 7;14:1099009. doi: 10.3389/fpls.2023.1099009 (PMC10028098; doi:10.3389/fpls.2023.1099009)
Supplement: Supplementary file 3 [file Table_2.docx]

| **Genotype** | **Arabinose** | **Galactose** | **Glucose** | **Xylose** |  |
| --- | --- | --- | --- | --- | --- |
| **B73** | 59.0±3.6 | 15.9±0.7 | 123.6±9.4 | 113.6±7.1 |  |
| ***cal1*** | 49.6±2.0** | 12.7±0.8** | 154.4±7.8** | 143.2±5.8** |  |
| ***cal2*** | 73.3±3.8** | 15.1±1.6 | 76.9±30.4* | 120.2±11.3 |  |
| ***bm1*** | 51.5±3.5* | 12.9±0.9** | 103.9±11.2* | 122.6±6.7 |  |
| ***bm3*** | 77.1±2.9** | 20.3±1.4** | 78.5±8.7** | 149.9±4.8** |  |
| **A619** | 67.6±1.1 | 15.6±0.3 | 132.4±15.8 | 127.1±9.8 |  |
| ***cal3*** | 62.7±4.3* | 14.0±1.2* | 38.4±9.8** | 136.0±8.9 |  |
| ***cal4*** | 71.6±4.8 | 15.8±1.1 | 80.3±13.7** | 122.2±3.5 |  |
| ***cal5*** | 67.1±1.9 | 14.5±0.5** | 43.9±10.3** | 117.4±11.0 |  |
| ***cal6*** | 55.5±4.1** | 11.8±0.8** | 100.9±10.8** | 127.2±5.8 |  |
| ***cal7*** | 64.4±3.4 | 14.0±0.7** | 81.4±7.7** | 129.6±12.3 |  |
| ***cal8*** | 66.5±7.9 | 14.9±2.5 | 77.1±8.8** | 132.2±2.4 |  |

#### **Supplementary Table 2.** **Absolute monosaccharide composition (**μg per mg dAIR) **of cal mutant maize seedlings**. All values are shown as the mean ± SD of each monosaccharide in of 5 biological replicates. cal1, cal2, bm1, and bm3 are in B73 genetic background. cal3, cal4, cal5, cal6, cal7, and cal8 are in A619 genetic background. Asterisk(s) indicate levels of statistical significance between each mutant and corresponding wild-type plant determined by two-tailed unpaired Student t-test at p-value < 0.01 (**), p-value < 0.05 (*).
