# Supplementary material for: A large-scale forward genetic screen for maize mutants with altered lignocellulosic properties
Source: Front Plant Sci. 2023 Mar 7;14:1099009. doi: 10.3389/fpls.2023.1099009 (PMC10028098; doi:10.3389/fpls.2023.1099009)
Supplement: Supplementary file 4 [file Table_3.docx]

| **Genotype** | **Mature tissue** | **Arabinose** | **Galactose** | **Glucose** | **Xylose** |  |
| --- | --- | --- | --- | --- | --- | --- |
| **B73** | leaf | 60.6±4.7 | 26.6±3.8 | 62.2±12.9 | 254.6±34.7 |  |
| ***cal1*** | leaf | 61.3±5.5 | 27.0±2.7 | 61.3±3.4 | 229.9±9.9 |  |
| ***cal2*** | leaf | 65.6±1.2* | 26.4±1.1 | 46.5±4.8* | 231.5±10.8 |  |
| ***bm1*** | leaf | 59.5±6.4 | 25.4±2.1 | 52.2±8.7 | 230.2±19.9 |  |
| ***bm3*** | leaf | 63.8±3.7 | 27.3±1.5 | 49.8±4.3 | 246.4±11.0 |  |
| **A619** | leaf | 53.2±4.9 | 20.7±2.3 | 33.9±5.1 | 208.5±21.8 |  |
| ***cal3*** | leaf | 63.4±3.7* | 24.6±1.7* | 36.8±3.9 | 231.0±12.5 |  |
| **B73** | stem | 34.5±3.7 | 10.9±1.5 | 25.8±1.4 | 227.2±9.0 |  |
| ***cal1*** | stem | 38.4±2.2 | 11.2±1.4 | 32.9±5.6* | 226.0±16.8 |  |
| ***cal2*** | stem | 55.8±6.0** | 12.7±1.5 | 25.1±2.9 | 223.9±3.8 |  |
| ***bm1*** | stem | 37.9±2.5 | 11.4±0.8 | 27.1±2.6 | 240.4±7.7* |  |
| ***bm3*** | stem | 37.9±5.7 | 10.6±1.7 | 21.9±3.5 | 238.7±17.7 |  |
| **A619** | stem | 49.9±3.3 | 13.1±1.6 | 22.2±2.1 | 216.2±10.0 |  |
| ***cal3*** | stem | 65.6±7.0* | 16.0±1.3* | 19.1±1.3 | 227.6±23.7 |  |

**Supplementary Table 3.** **Monosaccharide composition (μg per mg dAIR) of *cal* mutant mature tissues**. All values are shown as the mean ± SD (n ≥ 3) of each monosaccharide. *cal1*, *cal2*, *bm1*, and *bm3* are in B73 genetic background. *cal3* are in A619 genetic background. Asterisk(s) indicate levels of statistical significance between each mutant and corresponding wild-type plant determined by two-tailed unpaired Student *t*-test at *p*-value < 0.01 (**), *p*-value < 0.05 (*).
